# Supplementary material for: Human induced pluripotent stem cell-derived vocal fold mucosa mimics development and responses to smoke exposure
Source: Nat Commun. 2019 Sep 24;10:4161. doi: 10.1038/s41467-019-12069-w (PMC6760204; doi:10.1038/s41467-019-12069-w)
Supplement: Supplementary file 1 — Supplementary Information [file 41467_2019_12069_MOESM1_ESM.pdf]

Supplementary Information

Human induced pluripotent stem cell-derived vocal fold mucosa mimics development and responds to smoke exposure

Lungova et al.

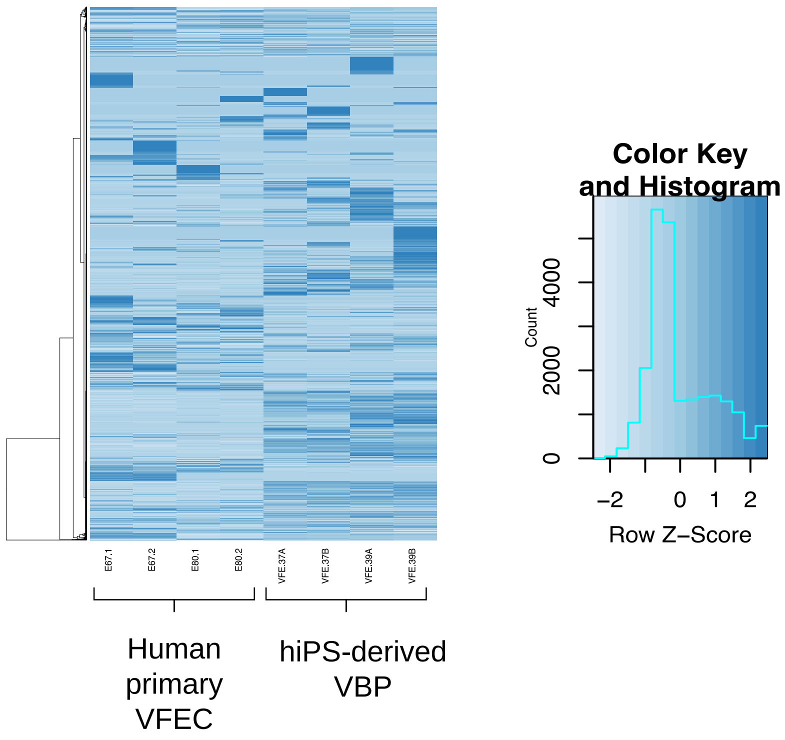

Supplementary Figure 1 RNA sequencing analysis. Heat map of equally expressed genes comparing human primary VF epithelial cells with hiPSC-derived VF basal progenitors. Abbreviations: VBP, vocal fold basal progenitors; VFEC, vocal fold epithelial cells.

a Top 10 Equally Expressed Genes

Index	Name	P-value	Adjusted p-value	Z-score	Combined score
1	G-protein coupled receptor signaling pathway (GO:0007186)	0.001581	0.1456	-4.85	9.35
2	nuclear-transcribed mRNA catabolic process, nonsense-mediated decay (GO:0000184)	1.720e-11	1.215e-8	-4.07	74.18
3	SRP-dependent cotranslational protein targeting to membrane (GO:0006614)	1.058e-13	1.121e-10	-4.02	92.08
4	rRNA processing (GO:0006364)	0.0001507	0.02904	-3.30	11.67
5	translation (GO:0006412)	0.000002203	0.0007780	-3.24	23.20
6	chemical synaptic transmission (GO:0007268)	8.947e-8	0.00003792	-3.08	31.38
7	translational initiation (GO:0006413)	3.318e-14	7.031e-11	-3.04	71.05
8	cornification (GO:0070268)	0.0005652	0.06654	-2.95	8.01
9	viral transcription (GO:0019083)	9.670e-11	5.122e-8	-2.80	46.97
10	cell surface receptor signaling pathway (GO:0007166)	0.003909	0.2528	-2.62	3.60

b Top 10 Differentially Expressed Genes

Index	Name	P-value	Adjusted p-value	Z-score	Combined score
1	collagen fibril organization (GO:0030199)	1.145e-11	1.147e-7	-3.32	83.57
2	extracellular matrix disassembly (GO:0022617)	1.181e-10	1.479e-7	-3.51	80.20
3	negative regulation of transcription, DNA-templated (GO:0045892)	0.000005548	0.001589	-6.61	79.94
4	peptidyl-tyrosine phosphorylation (GO:0018108)	3.345e-8	0.00001863	-4.49	77.21
5	positive regulation of transcription, DNA-templated (GO:0045893)	0.00001775	0.002578	-7.03	76.87
6	fibronectin fibril organization (GO:1905590)	4.703e-11	1.210e-7	-3.15	74.93
7	biofilm matrix organization (GO:0098784)	8.452e-11	1.210e-7	-3.11	72.16
8	cellulose microfibril organization (GO:0010215)	8.452e-11	1.210e-7	-3.11	72.13
9	extracellular matrix organization involved in endocardium development (GO:0061148)	8.452e-11	1.210e-7	-3.11	72.08
10	extracellular matrix organization in marginal zone involved in cerebral cortex radial glia guided migration (GO:0021820)	8.452e-11	1.210e-7		

Supplementary Figure 2 (a) Go biological processes analysis for top 10 equally expressed genes in human primary VF epithelial cells and hiPSC-derived VF basal progenitors. (b) Go biological processes analysis for top 10 differentially expressed genes in human primary VF epithelial cells and hiPSC-derived VF basal progenitors.

a

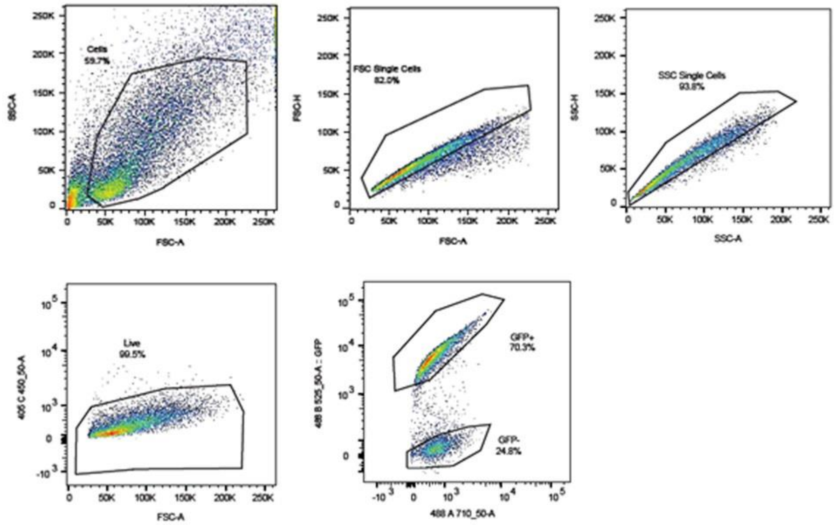

b

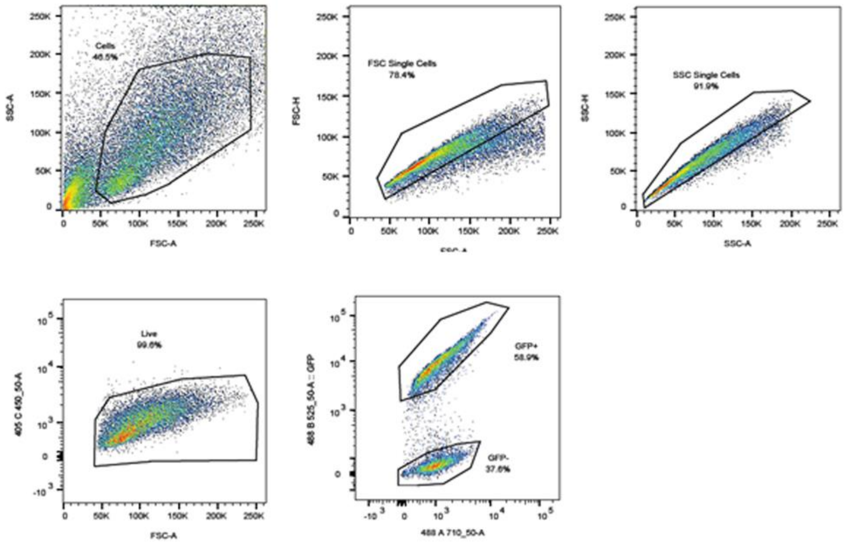

Supplementary Figure 3 (a, b) Sorting strategy for separation of GFP+ VFECs and GFP- VFFs from collagen gel in control samples (a) and from collagen gel after exposure to 5% CSE for 1week (b).

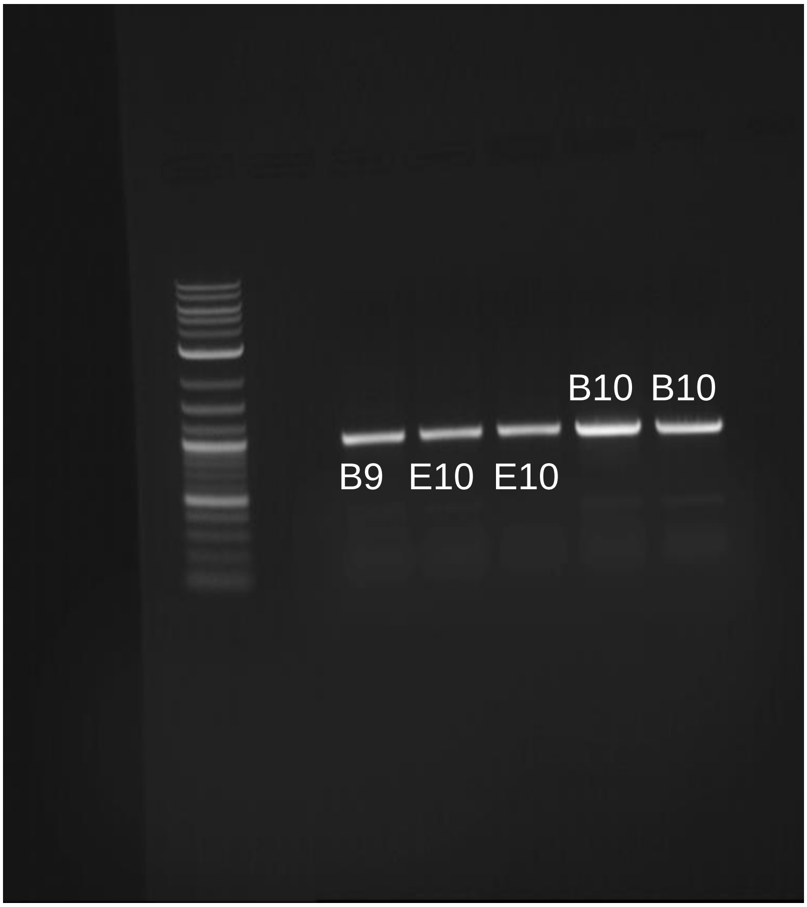

Supplementary Figure 4 The uncropped image of PCR analysis for correct GFP insertion into the genome of hiPS cells.

Supplementary Table 1

Genes and primer sequences

Gene	Forward (5’-3’)	Reverse (5’-3’)
5′-junction PCR	CTGCCGTCTCTCTCCTGAGT	GTGGGCTTGTACTCGGTCAT
INTERNAL AAVS1 PROBE	GGCCTGGGTCACCTCTACG	GAACCAGAGCCACATTAACCG
Sox2	GGAGAGTAAGAAACAGCATGGA	GTGGATGGGATTGGTGTTCT
Sox17	TCCTGGAGGAGCTAAGGAAA	ACTGTTCAAGTGGCAGACAA
FoxA2	GGCCAGAGTTCCACAAATCTA	CCCTCCCTCCTTCTTGAAATAAT
Tp63	CACCATGTGAGCTCTTCCTATC	AGGTAGCCTCTTACTTCTCCTT
Cytokeratin K8	CAGGAGCTGATGAACGTCAA	CATGTTCTGCATCCCAGACT
Cytokeratin K5	CTGCTCTACTGTGGGGTGTA	GAGTTGCTCAGGTGCTTGGA
Cytokeratin K14	AGTCCCTACTTCAAGACCATTGAG	GGTTCAACTCTGTCTCATACTTGG
Cytokeratin K13	TCAAGACACGTCTGGAGCAG	AAGTCAGACAGTGAGGGGTCT
SHH	GTCATCAGTTCCATGGGCGA	TTTTGGGGTGCCTCCTCTTC
Beta-Actin	ACGTTGCTATCCAGGCTGTGCTAT	CTCGGTGAGGATCTTCATGAGGTAGT
CDX2	TAAATGCCAGAGCCAACCTGACTTCC	CAGCAGCAACAACAACACAAACTCCC
CDH1	CGATTAAAGGTGGAGAGAGGACTG	AATGAATGGTGGACAGACACAGG
MUC1	AGACGTCAGCGTGAGTGATG	GACAGCCAAGGCAATGAGAT
MUC4	GGGAAGAAAGGCCCAACTAC	CTATGCTGACGGGTTGGAAT
IL6	AAGCCAGAGCTGTGCAGATGAGTA	GCTGCGCAGAATGAGATGAGTTGT
IL8	AGACATACTCCAAACCTTTCCACCC	TCCAGACAGAGCTCTCTTCCATCA
Col 1A1	GCAGACTGGCAACCTCAAGA	TGTGACTCGTGCAGCCATC
Col 1A2	AACAAATAAGCCATCACGCCTGCC	TGAAACAGACTGGGCCAATGTCCA
HAS3	TGTGCAGTGTATTAGTGGGCCCTT	TTGGAGCGCGCGGTATACTTAGTT
TGFbeta	TGCTCGCCCTGTACAACAGCA	CGTTGTGGGTTTCCACCATTAGCA
MMP2	AGAAGGATGGCAAGTACGGCT	AGTGGTGCAGCTGTCATAGGATGT

Supplementary Table 2

Enriched genes upregulated in the primary VF epithelium and hiPSC-derived VF mucosa or esophagus

Genes	Human primary VFE	iPS-derived VFE	Human fetal esophagus
**C1orf85**	2035	2132	525
**C22orf28**	2864	2387	818
**CARL**	35688	23361	7407
**CANX**	29307	24985	4357
**CDH13**	12953	5138	284
**FOXC2**	164	370	19
**KLF16**	685	705	24
**NME2**	7305	7117	24
**TCEA1**	3963	4650	660
**RHOC**	8124	8424	1999
**HOXB3**	325	168	1738
**PBX3**	476	582	1108
**PBX4**	1.9	10	133
**RFX3**	71	89	576
**SAMHD1**	426	589	2921
**MUC22**	16	9	38
**SOX13**	468	335	1696
**SOX2**	385	173	4712
**NOTCH4**	10	16	492
**CDH6**	231	59	853

Expression levels of enriched genes are average values calculated from two biological and two technical replicates in human primary VF epithelial cells n=4 and two biological and two technical replicates in hiPSC-derived VF mucosa isolated from two differentiation rounds n=4. Human esophageal data are average values calculated from four biological replicates n=4 obtained from the publically available database (24).
